# An extreme sea level indicator for the contiguous United States coastline

**DOI:** 10.1038/s41597-019-0333-x

**Published:** 2019-12-18

**Authors:** Md. Mamunur Rashid, Thomas Wahl, Don P. Chambers, Francisco M. Calafat, William V. Sweet

**Affiliations:** 1 Civil, Environmental, and Construction Engineering & National Center for Integrated Coastal Research, University of Central Florida, Orlando, FL USA; 2 College of Marine Science, University of South Florida, St. Petersburg, FL USA; 3 National Oceanography Centre, Liverpool, UK; 4 NOAA National Ocean Service, Silver Spring, MD USA

**Keywords:** Physical oceanography, Natural hazards

## Abstract

We develop an aggregated extreme sea level (ESL) indicator for the contiguous United States coastline, which is comprised of separate indicators for mean sea level (MSL) and storm surge climatology (SSC). We use water level data from tide gauges to estimate interannual to multi-decadal variability of MSL and SSC and identify coastline stretches where the observed changes are coherent. Both the MSL and SSC indicators show significant fluctuations. Indicators of the individual components are combined with multi-year tidal contributions into aggregated ESL indicators. The relative contribution of the different components varies considerably in time and space. Our results highlight the important role of interannual to multi-decadal variability in different sea level components in exacerbating, or reducing, the impacts of long-term MSL rise over time scales relevant for coastal planning and management. Regularly updating the proposed indicator will allow tracking changes in ESL posing a threat to many coastal communities, including the identification of periods where the likelihood of flooding is particularly large or small.

## Introduction

In the United States (U.S.), coastal counties contribute approximately 48% to the national Gross Domestic Product and 40% of the total population (https://coast.noaa.gov/states/fast-facts/economics-and-demographics.html)^1^. 17 major cities, each with populations of more than one million, lie in these counties; eight of them were listed in the top 20 of global major coastal cities in terms of present-day and future flood risk^2^. Impacts of coastal flooding to these densely populated and highly developed hotspots can be devastating, as exemplified recently during the 2017 and 2018 hurricane seasons with historical flooding in Houston, parts of Florida, and the Carolinas. Coastal flood risk is modulated by the ongoing global mean sea level rise as well as variability in mean sea level (MSL), storm surge climatology (SSC), and long-period tidal modulations. Expected flood impacts for coastal zones by mid-century will be substantial without aggressive greenhouse gas mitigation, combined with new and upgraded built and natural defenses^2,3^. Therefore, a detailed understanding of the primary oceanographic drivers of changes in coastal flood risk, MSL and SSC, at annual and longer time scales is crucial for developing sustainable adaptation plans, including the timing of when to implement them.

Coastal flood risk assessments often account only for long-term trends in MSL (regional or global)^3,4^ and SSC^5^. This ignores interannual to multi-decadal variations in both components that are driven by different mechanisms and can escalate or reduce flood risk over time scales from years to decades^6–8^. Accounting for these variations can lead to the exceedance of certain critical thresholds much earlier (or later) as compared to assuming smooth gradual changes, or it can lead to larger (smaller) changes than anticipated for given target periods in the future.

Variations in both MSL and SSC at seasonal to decadal time scales have been assessed for the U.S. coast^8–11^ and other parts of the globe^8,9^, usually separate from each other using a range of different analyzing techniques. The observed changes were coherent, in each of the variables individually, for long coastline stretches, suggesting that representative MSL and SSC indicators could be constructed for larger regions and that the fluctuations are linked to large-scale climate variability^6,9^. While there is still a need to examine MSL changes and SSC for individual tide gauges or localities, there are many regions where such an analysis is impossible due to a lack of observations. However, since both SSC and MSL variability are spatially coherent for large regions due to the specific dynamics driving the variability, an integrated, regional indictor for extreme sea levels (ESL) that includes both MSL and SSC variability for the U.S. coast would be a useful tool that can be exploited by the scientific community, in collaboration with stakeholders, planners, and engineers. Smaller communities in particular, where no observational records exist, can benefit from the information on regionally coherent variability in ESL and how it modulates return water levels typically used in risk assessments or design. But also at locations where longer tide gauge records exist, the information is useful, because of the regional coherence, and can be directly incorporated into local assessments.

Here we develop such an indicator using a comprehensive framework: we start with analyzing the MSL variability based on low-pass filtered monthly records of MSL from tide gauges along the contiguous U.S. coast, and develop regional indices, where variability is coherent (see Methods). For the SSC component, we use hourly water levels from tide gauges and apply quasi non-stationary extreme value analysis^11^ to quantify multi-decadal variability (see Methods). Cross-correlation analysis and k-means clustering are used to identify regions of coherent fluctuations. The regional indices of the two components are combined, along with time series representing tidal influences from the 4.4-year perigean and 18.6-year nodal cycles, into an aggregated ESL indicator. Long-term relative MSL rise can be added as an additional component, but here we focus on the interannual to multi-decadal variations around these long-term trends.

## Results

### Mean sea level indicator

**Fig. 1:**
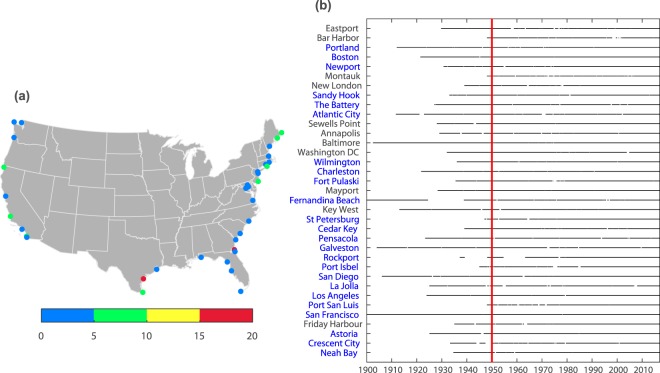
Geographical location and data information of selected tide gauges. Spatial presentation of selected tide gauges for the SSC analysis and their corresponding percentage of missing hourly values (**a**). Data availability of selected tide gauges for MSL and SSC analysis since 1900 (**b**). Red line represents the reference year 1950. Blue text represents tide gauges used for both SSC and MSL analysis, black text denotes tide gauges only used for the SSC analysis. Four tide gauges that do not provide long hourly records are only used for the MSL analysis and not displayed: Seattle, Grand Isle, Hampton Road, and Annapolis.

**Fig. 2:**
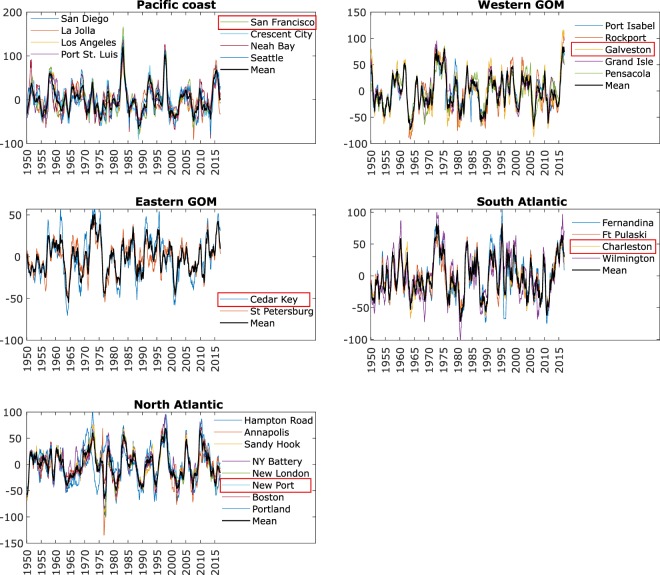
MSL indicator development. The interannual variability of mean sea level (in mm) over time for each tide gauge record in different regions along the U.S. coastline (colored lines), along with the statistical average (black line); tide gauges providing the longest records for each region and used as the MSL indicators hereafter are marked with red boxes.

28 monthly tide gauge records (Fig. 1) were processed as follows: influences of vertical land movement (VLM) were removed (see Methods), before the residuals were low-pass filtered with an annual running mean and grouped using cross-correlation analysis to identify regions of coherent 1- to 10-year variability (Fig. 2). At this stage we focus on the common time period from 1950 onwards which is covered by all records. However, some tide gauges provide significantly longer records than others, and we want our aggregated ESL indicator to cover the longest possible time period. Hence, after carefully identifying regions of coherent variability, we select the low-passed filtered time series of the tide gauge with the longest record (highlighted by a box in Fig. 2) as the MSL indicator for that region.

As in Thompson, *et al*.^10^, we find the interannual sea level at eight tide gauges on the Pacific coast (San Diego, La Jolla, Los Angeles, Port St. Luis, San Francisco, Crescent City, Neah Bay, and Seattle) correlated well with that measured at the other gauges on the coast (r > 0.65, p < 0.01), except at Astoria. The Astoria gauge is installed far upstream the Columbia River, and likely exposed to more riverine influences^11^, so was not included further in the analysis. 50% of the pairs have correlations >0.8, and 24% have correlations between 0.7 and 0.79. The percent variance explained (PVE) ranges from 50% to 93%. A simple average of the eight gauges correlates well with the individual gauge records and explains 75% to 86.0% of the variance, with an average of 80.3% and a standard deviation of 4.8%.

Although Thompson and Mitchum^12^ found significant correlation for sea level along the Gulf Coast and Atlantic coast when the data were low-pass filtered, the mean of the data only explained approximately 50% of the variance at any gauge. Our cross-correlation PVE analysis indicates that the eight tide gauges in the Gulf of Mexico should be grouped separately to the Atlantic coast. Additionally, our analysis suggests two clusters for the Gulf of Mexico, representing the western and eastern parts. Although the eastern gauges are significantly correlated with the western gauges (r > 0.6), the interannual sea level variance in the east is 40% of the western variability, and the PVE between St. Petersburg or Cedar Key and any of the western gauges is less than 40%. Thus, we treat them differently. The five western Gulf of Mexico gauges (Port Isabel, Rockport, Galveston, Grand Isle, and Pensacola) show minimum and maximum correlation coefficients of 0.91 and 0.95 with the average (PVE from 78% to 90%). The two records on the west coast of Florida (Cedar key and St. Petersburg) correlate with the mean with 0.96, 0.93, and the mean explains 90% and 85% of the interannual variance.

Key West, which has a record that extends back to 1913, did not fall into either the Gulf of Mexico or southeast Atlantic grouping. This is likely due to effects from the Loop Current, which passes between it and Cuba^13^. Using the Key West data in either the eastern Gulf of Mexico indicator or the southeast Atlantic indicator reduces correlations to below 0.8 in both regions and PVE below 41%. While still highly significant, we do not include Key West for the MSL indicator development.

A simple average of the four South Atlantic gauges (Fernandina, Ft. Pulaski, Charleston, and Wilmington) correlates with the individual records with a minimum value of 0.89 (Wilmington) and a maximum of 0.98 (Charleston). The percent variance explained ranged from 80% in Wilmington to 96% in Charleston. Although the southeastern Atlantic MSL variability is correlated with the eastern Gulf of Mexico indicator at 0.75 (p < 0.01), the variance of the southeastern Atlantic indicator is two times higher than that of the eastern Gulf of Mexico, and as a consequence only 56% of the variance in the southeastern Atlantic indicator can be explained by the eastern Gulf of Mexico indicator. Thus, we believe they should be treated as separate regions for the purpose of the development of the MSL indicator.

A simple average of the eight North Atlantic gauges (Hampton Road, Annapolis, Sandy Hook, NY Battery, New London, Newport, Boston, and Portland) correlates with the individual records with a minimum value of 0.73 (Portland) and a maximum of 0.97 (Newport). Percent variance explained ranged from 50% in Portland to 93% in Sandy Hook. Only Portland had a value lower than 81%.

**Fig. 3:**
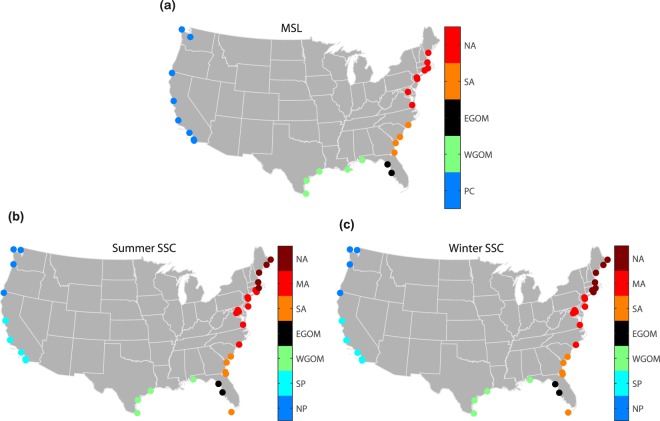
Regions of coherent MSL and SSC variability. Regions of coherent MSL variability (**a**) and SSC variability in the summer (**b**) and winter (**c**) half years (representing tropical and extra-tropical seasons) (PC: Pacific coast, NP: North Pacific, SP: South Pacific, WGOM: western Gulf of Mexico, EGOM: eastern Gulf of Mexico, SA: South Atlantic, MA: Mid-Atlantic, NA: North Atlantic).

Hence, for the MSL indicator we find five regions of coherent variability comprised of tide gauge records on the Pacific coast, western Gulf of Mexico, eastern Gulf of Mexico, south Atlantic, and north Atlantic (Fig. 3a).

### Storm surge climatology indicator

For the SSC indicator we analyze events in the extra-tropical season (or winter half year; November to April) separately from events that occurred during the tropical cyclone season (or summer half year; May to October) for 35 tide gauges along the contiguous U.S. coast (Fig. 1). We remove the influence of MSL (long-term trends and variability) and the effect of long period tides (4.4-year perigean and 18.6-year nodal cycles) on tidal high waters (see Methods). From the residuals, we identify the maximum still water levels in each season for a given year, i.e., we apply a seasonal block-maxima approach. Next, we identify seven regions of coherent variability using k-means clustering (see Methods): North Pacific (Neah Bay, Crescent City, Astoria, and Friday Harbor), South Pacific (San Francisco, Port San Luis, Los Angeles, La Jolla, and San Diego), western Gulf of Mexico (Pensacola, Galveston, Rockport, and Port Isabel), eastern Gulf of Mexico (St. Petersburg and Cedar key), South Atlantic (Key West, Fernandina Beach, Mayport, Fort Pulaski, and Charleston), Mid-Atlantic (Wilmington, Washington, Baltimore, Annapolis, Sewells Point, Atlantic City, NY Battery, Sandy Hook, New London, and Montauk), and North Atlantic (Newport, Boston, Portland, Bar Harbor, and Eastport) (Fig. 3c,d).

Hence, regions of coherent SSC and MSL variability overlap in the Gulf of Mexico, whereas on the Pacific coast two regions exhibit distinct SSC variability while MSL variability is coherent along the entire coast. On the Atlantic coast we identify three regions of distinct SSC variability but only two regions of distinct MSL variability.

We want the SSC indicator to reflect multi-decadal variability, and thus use the seasonal maxima discussed above to perform a quasi-non-stationary extreme value analysis (see Methods). This allows us to express the multi-decadal SSC variability as return water levels (RWL), for all tide gauges for the summer and winter seasons. As expected, the seasons that dominate RWL vary across regions. A season is considered dominant when RWL values are consistently higher for that season. For the North Pacific, South Pacific, and North Atlantic regions winter is the dominant season indicating that extra-tropical storms are more relevant, whereas for the Gulf of Mexico, South Atlantic, and Mid-Atlantic regions summer is the dominant season due to the influence of tropical cyclones. It is well known that the largest storm surges in the Gulf of Mexico and South Atlantic coasts are caused by tropical storms and hurricanes, whereas extra-tropical storms are responsible for the largest storm surges along the U.S. Pacific and North Atlantic coasts^14–16^. Our results support the decision to assess SSC variability separately for summer and winter seasons, even though for coastal design typically one RWL time series from annual maxima (or threshold exceedances) is considered. But this may not adequately capture storm surge variability associated with both tropical and extra-tropical cyclones. This is in support of findings by Orton, *et al*.^17^, who concluded that characteristics of coastal flooding hazards driven by the two types of storms are different due to different meteorology and frequency of the storm types, and they suggested to consider them separately in flood risk assessments. It also facilitates more robust assessments of future changes in coastal flood risk where climate change will likely affect tropical and extra-tropical storm characteristics differently^18–20^.

**Fig. 4:**
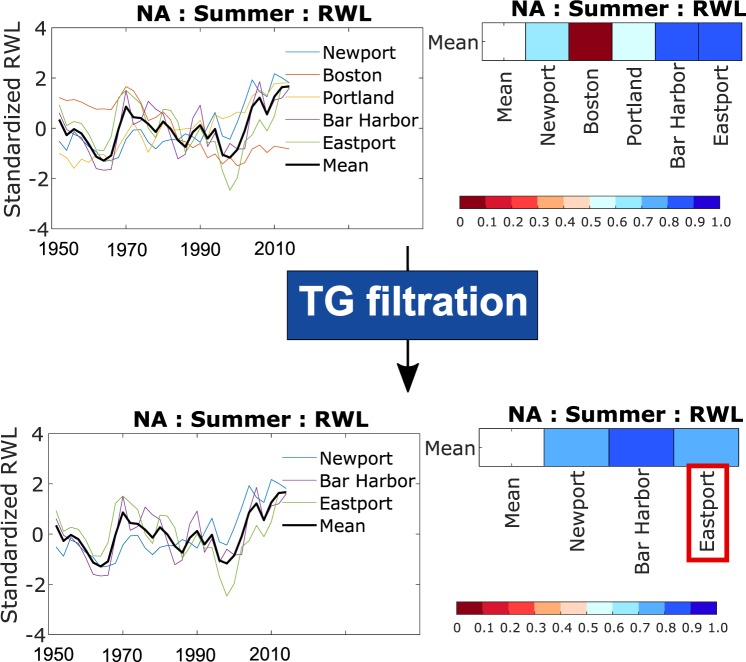
Filtration of representative tide gauge. Iterative filtration process to identify the representative tide gauge for the North Atlantic (NA) region in the summer season. Top: standardized RWL time series of all tide gauges within the region (left) for the common period of 1950 to 2017 and their corresponding correlation coefficient with the mean regional RWL time series (right). Bottom: same as the top panel but after removing tide gauges with correlation less than 0.6 with the regional average RWL time series; the representative tide gauge (i.e., the longest one of those retained through the filtration process) is highlighted in the bottom right panel.

**Fig. 5:**
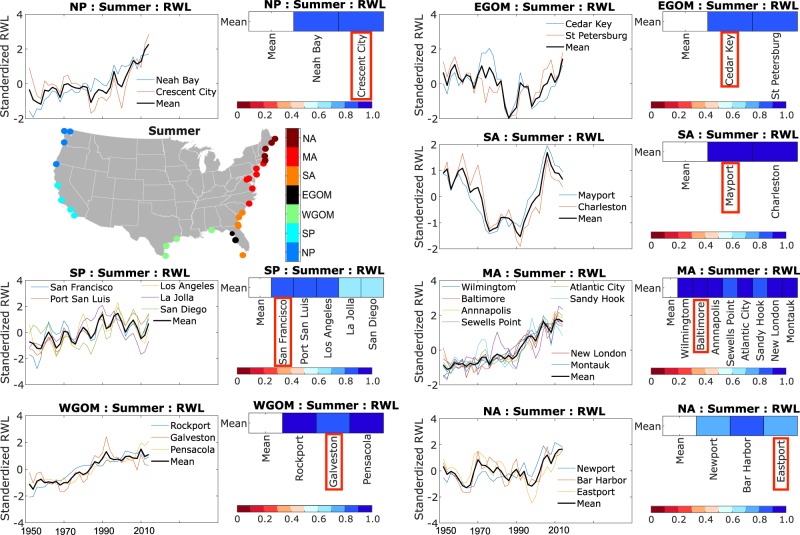
Regions of coherent SSC variability and corresponding representative tide gauges. Map showing regions of coherent SSC variability in summer and identification of representative tide gauges (providing the SSC indicator) for all regions using the filtration process described in the text considering the common period from 1950 to 2017 (x-axis).

**Fig. 6:**
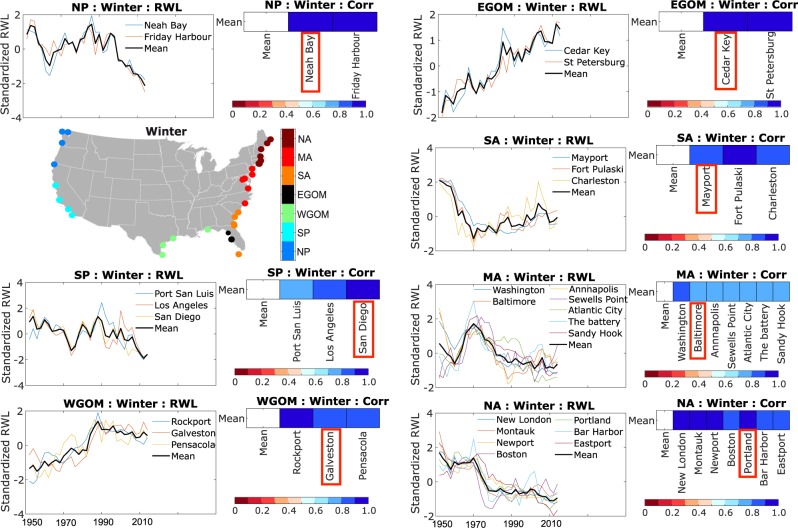
Same as Fig. 5 but for the winter season.

Regions of coherent variability of RWL time series (considering a common period from 1950 to 2017) overlap with the regions obtained from the k-means clustering analysis of the seasonal maxima values. This indicates that the spatio-temporal variability in the seasonal maxima persists in the RWL time series. To obtain the SSC indicator we examine RWL changes in each region separately with an iterative filtration process that retains only tide gauges with correlations above a certain threshold with the average regional RWL time series (which is re-calculated each time a tide gauge is removed; see Methods). Subsequently, we identify a representative RWL time series for each region that covers the longest period to be used as the SSC indicator. For the North Atlantic region, as an example, all five tide gauges within the region display a similar temporal pattern in the RWL time series (Fig. 4, top), but Boston and Portland show lower correlation with the average (most likely because of local effects related to freshwater inflow or changes in the high-frequency tidal constituents due to anthropogenic impacts) and are therefore removed. For Boston, as an example, secular changes in the amplitude of the M_2_ tidal constituent have been reported^21^, and attributed to tidal resonance and/or ocean stratification changes^22,23^. Of the remaining three RWL time series (Fig. 4, bottom) we select the one with the longest record, in this case Eastport, as representative for the region, and hence as the SSC indicator for the North Atlantic coast. We follow the same procedure to identify representative tide gauges and SSC indicators for all regions and seasons (Figs. 5 and 6).

**Fig. 7:**
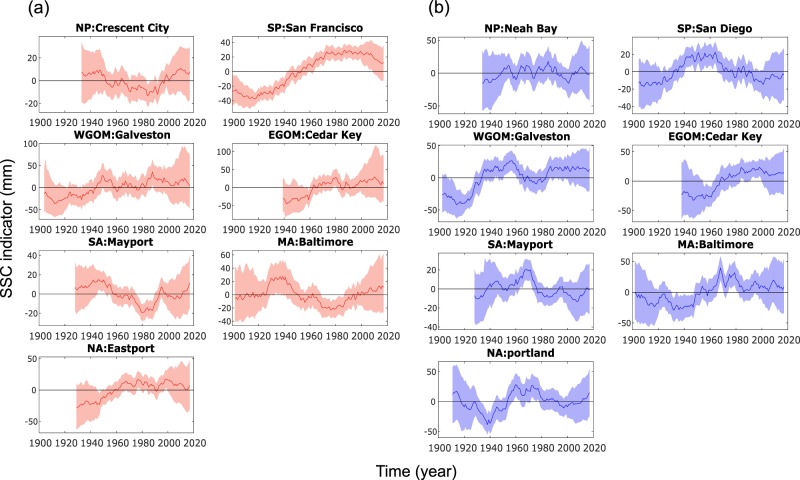
SSC indicators of different regions for summer and winter. SSC indicators representing RWL variability (in mm) around the mean and corresponding uncertainty bands (at the one-sigma level) for summer (**a**) and winter (**b**) seasons.

**Fig. 8:**
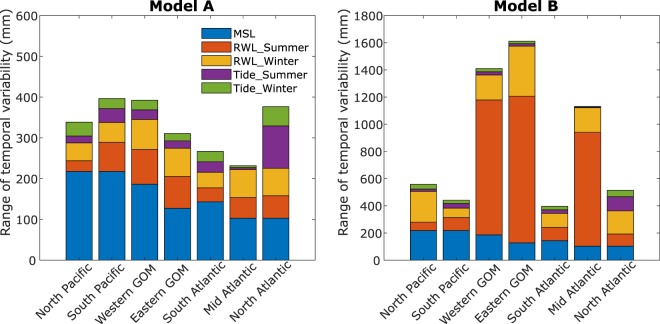
Range of variability of different components of the ESL indicator. Range of variability of long-period tides, MSL, and SSC indicators (the latter represented by time-varying RWL) for the different regions and from using Model A (left; only location parameter varies with time) and Model B (right; both location and scale parameters vary with time).

After selecting representative tide gauges for all regions, we derive RWL time series for the entire record lengths. Note, that we use the 100-year RWL here, but can chose any other return period instead through the time-dependent distribution parameters (see Data Availability section)^24^. Since RWL magnitudes (referred to a common datum) vary substantially for different regions of the U.S. coast, the temporal variability is explored based on the normalized RWL time series (i.e., the mean value over the entire RWL time series is subtracted) (Fig. 7). For the results shown here only the location parameter is considered non-stationary while scale and shape parameters are assumed constant. The range of temporal RWL variability is generally higher in the South Pacific, Gulf of Mexico, and Mid- and North Atlantic regions, compared to the North Pacific and South Atlantic regions. These differences in the range of RWL variability result not only from differences in storminess (including types of storms affecting different regions), but also from variations in the local coastal bathymetry, and orientation/shape of the coastline (e.g., shallow vs. deep water or wide vs. narrow continental shelf). Taking the South Pacific region as an example, the results in Fig. 7 indicate that in the summer season (San Francisco is the representative tide gauge) the 100-year RWL was up to 8 cm higher around the 2000s compared to the early 20^th^ century (unrelated to any changes in relative MSL), whereas in winter (San Diego is the representative tide gauge) the 100-year RWL was highest mid-century and 3 to 4 centimeters lower in the early 20^th^ and 21^st^ centuries. Such changes along the Pacific coast could be related to El Nino/La Nina effects or influences of the Pacific Decadal Oscillation (PDO)^6^ (note that more detailed exploration of the physical drivers of the observed changes is beyond the scope of this study and will be presented elsewhere). The range of variability increases further when a non-stationary GEV model is used that considers time varying location and scale parameters (Fig. 8 and Data Availability section)^24^, but uncertainties also increase.

### Aggregated extreme sea level indicator

We consider MSL and SSC indicators as independent assuming that variations in both are driven by different mechanisms. We also do not find any significant correlation between the MSL and SSC indicators for any of the regions or seasons. Therefore, the aggregated ESL indicator is derived by additively combining the MSL and SSC indicators with time series representing the long-period tidal signals associated with the 4.4-year perigean and 18.6-year nodal cycles. Coastal design and risk assessments usually consider RWL in one way or another (often the 100-year event), which can change through time, when a non-stationary approach is considered; but as outlined above, this typically only includes (linear) long-term changes, but ignores interannual to decadal variability. The aggregated ESL indicator presented here combines the 100-year RWL and its multi-decadal fluctuations (to reflect SSC variability) with underlying observed variations in MSL and multi-year tidal cycles. In this regard, the latter two function in a similar fashion as the non-stationary location parameter considered in the SSC analysis as they shift the 100-year RWL (used here as an example) upward/downward through time. Figure 8 shows the maximum range of variability of the different components, where both SSC (represented by 100-year RWL changes) and tidal components are displayed for summer and winter separately. The tidal influence is determined from the same tide gauges where the SSC indicators come from, and hence can be different for summer and winter, when two different tide gauges are selected as representative, but can also differ slightly when the same tide gauge is considered. In addition to the non-stationary extreme value analysis where only the location parameter varies with time (Model A), we also performed the analysis with a model setup that allows the location and scale parameters to change over time (Model B). The maximum range of variability of the individual components varies across regions. In most regions the MSL indicator dominates when Model A is used, but the SSC indicator becomes dominant when model B is used in most regions where tropical cyclones during the summer season are important (at least when the 100-year RWL is assessed); this is similar to the findings presented in ref. ^6^ (note, that by including a non-stationary scale parameter the uncertainties also increase).

**Fig. 9:**
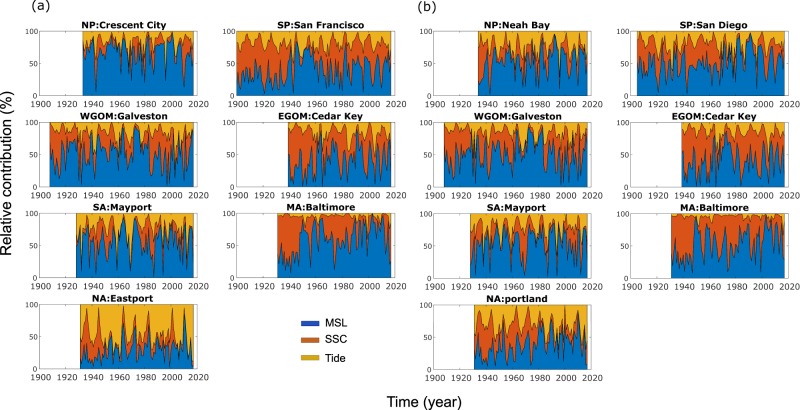
Relative contribution of different indicators to aggregated extreme sea level indicator. Relative contribution (in %) of MSL (blue), SSC (red), and tide (4.4- and 18.6 year) (yellow) to the aggregated ESL indicator in each region for summer (**a**) and winter (**b**) seasons. SSC indicators (i.e., 100 year RWL) were derived from GEV models where only location parameter varies with time.

Figure 9 shows that the relative contribution of the different components also changes over time (results are shown for Model A, but the same figure can be produced for Model B using the ‘sea level indicators’ hosted at figshare)^24^. All three components are more or less equally relevant for the South Pacific, Gulf of Mexico, and South Atlantic regions. In the North Pacific region, MSL is the largest contributor for most time steps. There are regions where the tidal contribution is very small (e.g., Mid-Atlantic) and other regions where it contributes most to interannual to multidecadal ESL variability (North Atlantic). The contribution of SSC (when represented by the 100-year RWL) is comparatively smaller in the North Pacific and South Atlantic regions, in both seasons. As outlined in Fig. 8, the SSC contribution increases significantly in some regions when Model B is used, allowing time varying location and scale parameters.

**Fig. 10:**
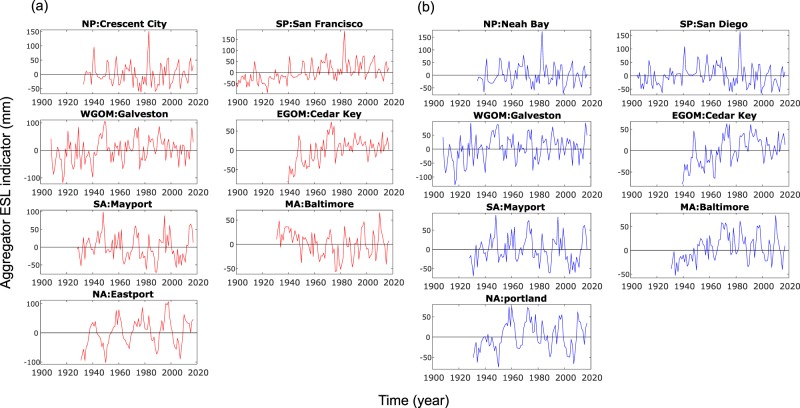
Aggregated ESL indicators. Aggregated ESL indicators (in mm) for the different regions and for summer (**a**) and winter seasons (**b**). SSC indicators represent variability in the 100-year RWL estimated from Model A, where only location parameters vary with time.

The aggregated ESL indicators displayed in Fig. 10 show significant variability over time, stemming from either of the three components discussed above. This highlights the potential implications of the variability represented by the indicators for coastal flood risk assessment and management, in addition to long term MSL trends. The latter is often assumed to be the only oceanographic driver for changes in coastal flood risk. The range of variability (i.e., max value minus min value) is in the order of one to three decimeters when Model A is used for the SSC indicator (when considering the 100-year RWL) and can reach more than a meter in some regions when Model B is used. The strong influence of multi-year tidal cycles (4.4-year and 18.6-year) on tidal high waters (and hence ESL) can be seen for the North Atlantic region, especially in the summer season.

## Discussion

We introduce a comprehensive framework using monthly and hourly tide gauge observations to estimate the interannual to multi-decadal MSL and SSC variability (the latter represented by temporal changes in 100-year RWL time series) for the contiguous U.S. coast. The aim of the indicator product presented here is to capture climate related variations in MSL and SSC, which show strong regional coherence as identified in earlier studies^25^, where such changes were also linked to large scale climatic influences^6^. This is confirmed in our analysis, and hence we first define regions of coherent variability and then estimate regional indicators by selecting a representative tide gauge that best explains the variability in the respective region.

Based on the analysis of regional coherence, two MSL indicators are developed for the Atlantic coast, with a separation approximately at Cape Hatteras, which is also the separation point of the Gulf Stream. The South Atlantic MSL indicator correlates with the North Atlantic MSL indicator with a value of 0.37. Although significant (p < 0.05), there are substantial differences between the two time series. In particular, while sea level has been dropping north of Cape Hatteras, it has been rising south of Cape Hatteras since 2010 (see Fig. 2). This has been noted by several authors^13,26–28^, who speculate that it is caused by changes in the strength of the Gulf Stream/Atlantic Meridional Overturning Circulation^13,26^, or the North Atlantic Oscillation and/or El Niño^27,28^. Whatever the dynamical reason, it is clear that two distinct MSL indicators are warranted.

When we identify the representative tide gauges for different regions we often find several tide gauges that are highly correlated, whereas others show different temporal patterns; this is more pronounced in the SSC analysis than in the MSL analysis. Examples are Boston (North Atlantic region) for the summer season, and Port Isabel (western Gulf of Mexico region) and Key West (South Atlantic region) for both seasons. Similar discrepancies were found in earlier studies^9,25^ and can be the result of differences in the local morphology/bathymetry (including anthropogenic modifications affecting tidal propagation), orientation and shape of the coast, or location of the tide gauge (e.g., in estuaries affected by freshwater or surrounded by coastal protection), although storm activity is the same for the entire region. Investigating each tide gauge and possible reasons for the discrepancies separately would be a useful exercise in the future, but is beyond the scope of the present study. Here, we find for all regions a number of tide gauges displaying very similar MSL and SSC variability, providing confidence that the signals we extract and use for the aggregated ESL indicator are the ones we are interested in, and no artifacts. Tide gauges where local characteristics lead to differences in the inter-annual to multi-decadal variability (e.g., changes in higher-frequency tidal constituents) are identified through the filtration process and dropped from the analysis. Furthermore, it has been shown in Wahl and Chambers^25^ that results from analyzing multi-decadal SSC variability at tide gauges that show coherent variations with others in the same region are insensitive to using still water level (as we do here), non-tidal residual (or surge), or skew surge time series.

Overall, we find variability at inter-annual to decadal time scales with ranges of up to a decimeter in the individual components and several decimeters when combined into the aggregated ESL indicator. The range of variability increases further when both location and scale parameters are allowed to change in the non-stationary extreme value analysis (Model A vs Model B in the SSC analysis). MSL and SSC display coherent variability within certain regions along the coast but distinct differences across regions, and, in case of SSC, also across seasons due to different storm types dominating the SSC variability. The combined results highlight the importance of inter-annual and decadal MSL and SSC variability in modulating ESL, and hence coastal flood risk. This can include an increase or decrease compared to the conclusions that one would draw when only considering smooth long-term (global average or regional) MSL projections as the sole oceanographic driver of changes in coastal flood risk.

A limitation of our study is that the number of available tide gauge records reduces significantly when going back in time. More tide gauges with longer records would allow more accurate estimation of multi-decadal variations and a geographically more precise separation of regions with coherent variability. Based on our selection criteria (see Methods) we use 28 tide gauges for the MSL analysis and 35 for the SSC analysis. This makes it difficult to identify the exact separation points between neighboring regions, as there are sometimes long coastline stretches in between with no (or insufficient) data. This can be improved by filling these spatial gaps using model hindcast data. For SSC, for example, the global tide and surge reanalysis (GTSR)^29^ provides a good starting point. However, the record length of 35 years (i.e., 1979 to 2014) is too short to quantify and analyze multi-decadal variability. Furthermore, models include additional uncertainties and may not capture all the processes responsible for the variability discussed and displayed above.

In summary, we have developed the first aggregated ESL indicator for the contiguous U.S. coast, comprised of separate indicators for MSL and SSC, as variability in both is different and driven by different mechanisms. The new indicator allows tracking the rise and fall in ESL relative to long-term trends and to identify periods in the past when flood risk was escalated or reduced over prolonged periods of time. This is the first step toward better understanding the causes of these fluctuations, by investigating links to large-scale climate variability, and to ultimately predict them, for example by using initialized decadal climate model simulations and downscale them. This would result in a step change in current practices in coastal flood risk assessment and management where (uncertain) long-term projections in MSL or SSC are usually the only drivers considered. While our study focuses on the U.S. coastline, the general framework can be applied to develop similar indicators for other countries or continents where suitable observational data (or results from model hindcasts) exist.

## Methods

### Data selection and processing

For the MSL analysis we use monthly sea level records obtained from the Permanent Service for Mean Sea Level (PSMSL, http://www.psmsl.org) from stations going back to at least 1950 and having few or no gaps. For the SSC analysis we use hourly sea level observations obtained from three different sources: University of Hawaii Sea Level Center (UHSLC; http://uhslc.soest.hawaii.edu/data/download/rq) database, Global Extreme Sea Level Analysis (GESLA; http://gesla.org/) database, and National Oceanic and Atmospheric Administration (NOAA; http://tidesandcurrents.noaa.gov/) water level database. The latter provides the most complete spatial and temporal coverage of hourly sea level data for the U.S. coast and was used as the primary data source. The other two sources (i.e., UHSLC and GESLA) were used to complement the NOAA data. Of the 121 tide gauges for the U.S. coast, we only considered the ones with records starting in 1950 or earlier and with maximum 20% of missing data; applying these criteria reduced the number of tide gauges to 45. Of these, 10 are located on Pacific Islands and hence outside our study region. Therefore, we considered 35 tide gauges with long-term hourly records for the SSC analysis, more than any other previous study where SSC variability was assessed for the U.S. coastline.

### Development of the mean sea level indicator

In order to develop the MSL indicator for interannual to decadal periods (~10–25 years), we first need to remove the influence of long-term MSL rise and vertical land motion (VLM), accounting for the fact that both may be non-linear (i.e., accelerating or decelerating). It is expected that users will add the resulting MSL indicator to any MSL trends, accelerations, or VLM that is appropriate for the study region. Previous studies examining interannual to decadal MSL variability have either fit and removed a linear trend, or linear trend after removing a linear trend of VLM from short Global Positioning System (GPS) colocations^16,20^. These methods assume that the trend of VLM measured by a GPS receiver is the same in the past and that the motion is linear, which may not be the case at many of the sites considered here (see additional file)^30^. Here, we choose to use a Gaussian weighted average filter with a rolloff of 10-years to estimate the combination of long-term sea level rise and VLM. The exact weighting function used is

$${\rm{weight}}\,({\rm{\Delta t}})={\rm{\exp }}\,(-0.5\,{({\rm{\Delta t}}/{\rm{rolloff}})}^{2}),$$

where Δt is the time from the center-point in the filter. The factor of 0.5 is used so that the weights drop to e^−1/2^ (0.6) when Δt = roll-off, instead of e^−1^ (0.36). Data for Δt = ±30-years from the central point is used in the calculation. This function will effectively damp all signals with periods below 25-years and pass through signals with >90% power at periods >35-years. The filter will estimate linear as well as non-linear and very-long-period (>25-years) signals in the data. The filter is applied after fitting and removing a trend and annual and semi-annual sinusoids (to reduce potential aliasing of these periodic fluctuations), then the trend is restored to leave the complete very-low-frequency (periods >25-years) MSL, including any trends and accelerations. Although this low-pass-filtered time series will also contain any multi-decadal variability (>25–30 year periods), this peaks at an order of magnitude smaller than the interannual to decadal variability^9^ of interest in this study, and many gauges with high linear VLM exhibit large, multi-decadal fluctuations that do not appear at other gauges in the region (see additional file)^30^. Also, removing only a linear trend results in less agreement between the gauges (see additional file)^30^. We assume these non-coherent very-low-frequency signals are most likely due to non-linear subsidence or uplift that is impossible to extract from a short, modern-day GPS record, as well as non-linear accelerations in long-term sea level rise.

After removing the very-low-frequency MSL from all sea level records, we computed a seasonal climatology at each site by averaging all January values, February values, etc. The climatology was removed and we focus on the determination of an interannual mean sea level index for each region. This is computed by applying a Gaussian weighted average filter (same form as before) with a roll-off of 6-months to the time-series, using a window of ±18 months from the central point. This filter suppresses all variability with periods less than 1-year and passes through all power in the spectra with periods >1.5 years. Periods between 1–1.5 years are attenuated at about 70% at 1.3 years, 50% at 1.4 years, and <10% at 1.5 years and above. Only data from 1950 onwards were used at this stage, as this is the common timeframe for all the data. These low-pass filtered time-series were then compared to group data into regions where all gauges had similar variability. Although this is often done using cross-correlations (*r*), this can over- or under-estimate how well the gauges agree, since the calculation does not retain variance. A better calculation is percent variance explained (PVE):

$$PVE=100\cdot \left(1-\frac{variance(tg1-tg2)}{variance(tg1)}\right)$$

where tg1 and tg2 are two different low-pass filtered tide gauge time-series. This calculation preserves the variance of the datasets and provides a more accurate computation of PVE. Note that unlike correlation, PVE can change slightly depending on which gauge is treated as tg1 (due to the differences in variance). Thus, we compute this for each pair both ways, and kept the larger value. We grouped tide gauges into coincident regions that explain at least 50% of variance of all pairs (equivalent to correlation of 0.7 if variances are identical). These grouped time-series are then averaged. For computation of the PVE of each tide gauge with the average, the same equation was used, simply substituting the average time series for tg2.

This analysis is performed to find the general coherence and average time series shown in Fig. 2. However, in order to aggregate with the ESL indicator and have the longest possible time-period, the time series from the tide gauge in each region that was most correlated with the average and had the longest record was selected as the indicator for the particular region. The time-series was simply averaged over each year (after removing the local climatology) without applying the 6-month Gaussian filter, as the ESL indicator was aggregated in terms of seasonal (not monthly) values.

### Development of the storm surge climatology indicator

For all selected 35 tide gauges, we extracted seasonal (summer and winter half of the year) extreme sea levels from the hourly water level observations. First, we removed the influence of MSL rise and variability by subtracting a 30-day running median at each time step from the hourly data. Next, we removed the low frequency tidal signals associated with the 4.4-year perigean and 18.6-year nodal cycles as these modulate high water levels^31^. We used the method employed by Haigh *et al*.^31^ and Ray *et al*.^32^, i.e., hourly time series of perigean and nodal cycles were estimated for each tide gauge using a harmonic fit to time series of the annual standard deviation of predicted tide time series obtained from a year-by-year harmonic tidal analysis conducted with T-Tide^33^. From the residuals (i.e., after removing MSL and multi-year tidal influence), we identified the highest values in the tropical (May to October) and extra-tropical (November to April) seasons. We excluded years where more than 25% of the hourly data were missing.

We employed a running window non-stationary extreme value analysis to identify the variability of SSC separately for summer and winter. We considered 37-year moving windows (i.e., twice the 18.6-year nodal cycle) centered at each time step and fit a Generalized Extreme Value (GEV) distribution to the seasonal maxima time series. Distribution and uncertainty parameters are estimated using the widely applied Maximum Likelihood Method. Following this approach we cannot directly estimate distribution parameters for 18 time steps (i.e., half of the selected window length) at the boundaries of the time series. To overcome this issue we employ a boundary padding technique widely used in wavelet decomposition and image processing. While symmetrization would be a straightforward way of boundary padding, we use a resampling Monte Carlo scheme to incorporate the uncertainties resulting from the boundary padding in our analysis. Boundary padding includes the following steps: (1) fit a GEV distribution to the seasonal maximum water level data and estimate the distribution parameters; (2) generate random seasonal maximum water level data using the distribution parameters obtained in step (1) for boundary padding. For example, an original seasonal maximum water level time series of 100 data points will be extended to include 136 data points (18 more at the beginning and end); and (3) estimate time varying location (and scale) parameters using the running window approach. Steps (2) to (3) are repeated 1,000 times to quantify the uncertainty introduced by the boundary padding (note, that only the boundary information is used from the Monte Carlo approach; in between, where data is available for the full length of the windows we use the original observations).

We considered two different model setups, the first one only assumes a non-stationary location parameter (Model A), the second one assumes non-stationary location and scale parameters (Model B); in both models the shape parameter was kept constant due to its highly sensitive characteristics. Finally, the GEV parameters estimated for each window are used to estimate 100-year RWLs to represent SSC variability; we provide the non-stationary distribution parameters in the ‘sea level indicators’ data hosted at figshare^24^ so that any other return period can be chosen to construct the SSC indicator and combine with the MSL and tidal components. A sensitivity test was carried out by fitting a Generalized Pareto distribution (GPD) to seasonal high water levels above a given threshold (instead of using seasonal maxima and the GEV distribution). The results displayed similar trend and variability patterns, which prompted us to use the GEV approach (avoiding subjective choices regarding the threshold selection for the individual tide gauge records).

We used k-means clustering to identify the regions of coherent SSC variability along the U.S. coast. As record lengths vary across tide gauges, we focus on the period from 1950 onwards for the regionalization. In order to avoid tide gauges from different coastlines to be attributed to the same cluster(s) (which happened only in few instances because of weak cross-correlation), we first subdivided the coast into three different regions: Pacific, Gulf of Mexico, and Atlantic coasts. From previous studies it is known that across these regions the dominant storm types (tropical vs extratropical), surge magnitudes, and corresponding climate situations are different^6,14,25,34,35^. We performed k-means clustering for the tide gauges within each of the three regions separately. This part of the analysis was complemented with a cross-correlation analysis to ensure that temporal co-variability and spatial proximity among the tide gauges of the clusters is optimized.

Finally, for each cluster (or region) we identified a representative tide gauge for the tropical and extra-tropical seasons from standardized RWL time series (i.e., deduct the mean and divide by the standard deviation of the entire RWL time series). The selection process includes the following steps: (1) calculate average of the RWL time series of all tide gauges within a region; (2) compute the correlation of the RWL time series from all tide gauges in the region with the mean RWL time series; (3) remove RWL time series from individual tide gauges showing correlation with the mean RWL time series below 0.6, (4) with the rest of the tide gauges estimate average of the RWL time series and compute correlation with RWL time series of the remaining tide gauges; (5) repeat steps (3) to (4) until all remaining tide gauges have correlation above 0.6 with the updated mean RWL time series; and (6) consider the tide gauge (among the ones retained after the filtering) with the longest record as representative for SSC variability in the tropical or extra-tropical season for the region. The latter are used as the SSC indicators. For this filtration process we consider the common period from 1950 to 2017 to include all tide gauges. In order to test the robustness of the analysis against the chosen time period we repeated the analysis for summer in the South Pacific (SP) region (where all tide gauges have long data) for the extended period from 1900 to 2017, which confirms the selection of San Francisco as the representative tide gauge for this season and region (see Fig. S3 and associated text in the additional file)^30^.

### Development of aggregated extreme sea level indicator

We developed aggregated ESL indicators for the seven regions with coherent SSC variability, as these were geographically more refined than the regions of coherent MSL variability. We linked the Pacific coast MSL indicator to both the North and South Pacific SSC regions, and the North Atlantic MSL indicator to the North and Mid-Atlantic SSC regions. Annual time series of MSL and SSC indicators and long-period tidal contributions (i.e., the combined 4.4- and 18.6-year cycles) were superimposed to derive the aggregated ESL indicators for summer and winter seasons. Finally, we calculated the maximum range of variability of the different components found in the entire record lengths, and also quantified the relative contribution of each component to the aggregated ESL indicators.

## Data Availability

The datasets generated and/or analysed during the current study are available in the *Figshare* data repository^24^. Additional files accompany this paper^30^.

## Code availability

Codes/scripts used in this study for data processing and analysis were developed in Matlab 2017b. Codes/scripts are stored in the *Figshare* data repository and can be downloaded from the link^36^.
